# MIDO COVID: A digital public health strategy designed to tackle chronic disease and the COVID-19 pandemic in Mexico

**DOI:** 10.1371/journal.pone.0277014

**Published:** 2022-11-17

**Authors:** Hector Gallardo-Rincón, Julieta Lomelín Gascon, Luis Alberto Martínez-Juárez, Alejandra Montoya, Rodrigo Saucedo-Martínez, Ricardo Mújica Rosales, Roberto Tapia-Conyer

**Affiliations:** 1 Carlos Slim Foundation, Mexico City, Mexico; 2 Health Sciences University Center, Guadalajara University, Guadalajara, Mexico; 3 London School of Hygiene and Tropical Medicine, London, United Kingdom; 4 National Autonomous University of Mexico, Mexico City, Mexico; National Institute of Perinatology: Instituto Nacional de Perinatologia, MEXICO

## Abstract

Screening, prevention, and management of non-communicable diseases (NCDs, including obesity, hypertension, and type 2 diabetes) is the core function of Integrated Measurement for Early Detection (MIDO), a digital strategy developed by the Carlos Slim Foundation in Mexico. An extension of this strategy, MIDO COVID, was developed to address the need for an integrated plan in primary health care during the COVID-19 pandemic. MIDO COVID facilitates planning, surveillance, testing, and clinical management of SARS-CoV-2 infections and the major NCDs and their pre-disease states, to streamline the continuum of care. MIDO COVID screening was applied in 1063 Carso Group workplaces in 190 municipalities of the 32 Mexican states. Staff were trained to screen healthy workers for NCDs using a questionnaire, anthropomorphic measurements, and blood work; healthy individuals returning to work also received a SARS-CoV-2 antibody test. Between June 26 and December 31, 2020, 58,277 asymptomatic individuals underwent screening. The prevalence of obesity, hypertension, and type 2 diabetes was 32.1%, 25.7%, and 9.7% respectively. Only 2.2%, 8.8%, and 4.5% of individuals, respectively, were previously aware of their condition. Pre-obesity was identified in 38.6%, pre-hypertension in 17.4%, and prediabetes in 7.5% of the population. Risk of SARS-CoV-2 infection was highest for individuals with multiple NCDs. Many Mexicans are unaware of their health status and potentially increased risk of COVID-19 and serious complications. As a universal strategy implemented regardless of social factors, MIDO COVID promotes equity in access to health care prevention and early stage detection of NCDs; the information gained may help inform decisionmakers regarding prioritising vulnerable populations for immunisation.

## Introduction

In Mexico, an increasing prevalence of chronic, non-communicable diseases (NCDs) such as obesity, hypertension, and diabetes has been reported in recent years [1, 2]. There is a strong relationship between the presence of such NCDs and COVID-19 severity and mortality [3–5]. According to Mexico’s National Dashboard for COVID-19, the most common co-morbidities among 2,169,007 positive cases reported as of March 17, 2021, in descending order, were hypertension (17.32%), obesity (14.46%), and diabetes (13.35%) [6]. Among 195,119 COVID-19-related deaths reported, hypertension was present in 45.22% of patients, obesity in 37.43%, and diabetes in 22.03% [7]. Poor metabolic control, i.e., hyperglycaemia, has been reported to be consistently associated with poor COVID-19 prognosis [8]. Therefore, early intervention and optimal glycaemic control are important for improving outcomes in patients with diabetes who become infected with SARS-CoV-2. Early identification and management of NCDs and risk factors are important to decrease COVID-19 severity and mortality.

In Mexico, there is a separation between agendas for the attention of NCDs and infectious diseases; i.e., primary care tends to focus on the initial reason for the consultation rather than taking a comprehensive approach to the overall needs of the patient. This fragmented approach contributes to a lack of effective communication between health care providers, and the gap in continuity of care contributes to unfavourable outcomes for patients. Barriers to accessing primary care in Mexico further exacerbate this, as the lack of resources (material, financial, and trained healthcare providers) and language barriers (in rural populations) contribute to difficulties in patients accessing care [9]. As a result of the COVID-19 pandemic, health care systems, including primary health care services, worldwide and in Mexico, have become partially or totally saturated, and the ability to attend patients with NCDs has been significantly reduced or suspended [10]. Therefore, there is a need to find strategies (including materials and finances) to strengthen health care services to be better prepared and equipped to attend, prevent, diagnose, and manage NCD cases, particularly in a pandemic context. An integrated person-based strategy for managing NCDs and infectious diseases at the first level of care is warranted.

According to its name in Spanish, the Integrated Measurement for Early Detection (MIDO) is an innovative digital strategy developed in Mexico in 2010 to screen for, prevent, and manage NCDs [11]. Between 2014 and 2018 in Mexico, 743,000 individuals were screened using MIDO, revealing that 89.8% had a pre-disease (e.g., high fasting plasma glucose, pre-obesity, or pre-hypertension) or disease (e.g., diabetes, obesity, or hypertension) condition. Most of these people (74.4%) were unaware of their diagnosis, and very few (5.47%) pursued subsequent timely care at a primary health centre [12].

MIDO COVID is a simple, comprehensive, person-centred digital strategy that responds to the need for an integrated plan in primary health care during the COVID-19 pandemic. Originally developed for application in the workplace, it has an educational component for medical and non-medical personnel, making it potentially useful to implement at health care centres and in social gathering areas. It facilitates planning for surveillance, testing, and clinical management of SARS-CoV-2 infections, pre-disease states, and NCDs related to COVID-19 severity and mortality. It also facilitates the application of the 3Ts (test-track-trace) World Health Organization (WHO) strategy and allows a saturated health care system to perform and streamline its continuum of care. MIDO COVID generates 21 digital health profiles that provide specific recommendations for preventing or controlling chronic conditions and COVID-19. This strategy focuses on five main axes: (1) prevention and timely detection of weight-related problems (overweight and obesity); (2) hypertension; (3) type 2 diabetes; (4) prevention, timely detection, and modification of the risk of SARS-CoV-2 infection; and (5) rapid serological testing for the detection of immunoglobulin (Ig) G and IgM antibodies to SARS-CoV-2. Finally, through the strategy *“Mi Coach de Salud”* (“My Health Coach” in English), MIDO COVID provides each person with recommendations for changes in lifestyle according to their health profile. MIDO COVID is intended to improve cost efficiencies through a comprehensive approach to preventive health care services in the context of increasing rates of NCDs in Mexico.

The objectives of this study were to investigate the disease profile of the population screened using MIDO COVID in Mexico, and to identify diagnosis gaps in the continuum of care from screening to control of the main NCDs. We also aimed to pre-emptively identify individuals exposed to SARS-CoV-2 before they became ill to enable early control of the disease, improve patient outcomes, and prevent outbreaks.

## Materials and methods

### Implementation of the MIDO COVID screening protocol

All individuals to whom MIDO COVID is applied have a unique “Monitor” identification, which allows for monitoring and reporting of symptoms during the COVID-19 pandemic [11]. The MIDO COVID screening protocol has three main implementation steps (**S1 File**). First, each participating company selected the staff responsible for implementing the MIDO COVID screening protocol in their respective workplace through their Human Resources Department (MIDO Expert). Each of the chosen staff underwent an online training course and onsite training with medical staff. Second, healthy workers returning to the worksite underwent testing for SARS-CoV-2 antibodies and chronic diseases, and epidemiological protocols were followed whenever a positive COVID-19 case was detected. Third, ongoing follow-up meetings were held with the head of Human Resources at each workplace and the MIDO COVID staff to address any questions or issues arising during the implementation of the screening protocol, to continue training initiatives, and to evaluate the plans or strategies implemented by the companies to care for the health of their workers. The main goal of implementing this screening protocol was to strengthen permanent health care programs in the workplace.

MIDO COVID screening was applied between June 26, 2020 and December 31, 2020 in 1063 workplaces of the Carso Group in 190 municipalities of the 32 states in Mexico. MIDO COVID was proposed by the Carlos Slim Foundation and implemented as a workplace protocol. The individuals screened were interviewed by the MIDO Experts at each workplace using the MIDO COVID questionnaire to assess the individuals’ medical history and smoking, exercise, and sleep habits. A copy of the questionnaire is available in the **S2 File**. MIDO COVID also collects demographic data on individuals, health status measurements, and serological test results. This study was conducted in accordance with the principles of the Declaration of Helsinki and ethical approval was waived as only blinded datasets of administrative records were analysed. All patients who underwent MIDO COVID screening provided verbal consent prior to participation, and were informed that their de-identified information would be made available for scientific analyses. The full, de-identified (patient names and identifiers removed) dataset is available in the **S3 File**.

### Study population

The study population comprised 58,277 workers of the Carso Group, a global conglomerate company with diverse involvement across the industrial, commercial, infrastructure, and telecommunications sectors. MIDO COVID screening was applied to all asymptomatic individuals in the workplace and non-pregnant women (pregnant women received normal standard of care as usual).

### MIDO screening measurements and disease profile definitions

Weight was measured in kilograms using a Detecto Pro-Doc 101 scale (Cardinal/Detecto, Webb City, MO, USA), height was measured in metres using a Detecto DHR-model stadiometer, and waist circumference was measured in centimetres at the midpoint between the inferior margin of the rib cage and the iliac crest using a SECA flexible tape measure (Seca GmbH, Hamburg, Germany). Body mass index (BMI) was classified according to WHO and US Preventive Services Task Force criteria [13]. Abdominal obesity was defined as waist circumference >102 cm in men and >88 cm in women, according to the Association for Weight Management and Obesity Prevention [14]. Blood pressure was measured after 5 minutes of rest using a Mindray VS-800 vital sign monitor (Shenzhen Mindray Bio-Medical Electronics Co., Ltd.), and hypertension was diagnosed according to the Joint National Committee on Prevention, Detection, Evaluation, and Treatment of High Blood Pressure criteria [15]. The following laboratory tests were performed: fasting and non-fasting capillary glucose and SARS-CoV-2 serological testing for IgG and IgM antibodies, to differentiate between current and previous infection.

Pre-disease states were defined as follows: pre-obesity/overweight, BMI 25–30 kg/m^2^; pre-hypertension, systolic blood pressure 130–139 mmHg and diastolic blood pressure 85–89 mmHg; and prediabetes, fasting plasma glucose 5.6–6.9 mmol/L (100–125 mg/dL), 2-h post-prandial plasma glucose 7.8–11.0 mmol/L (140–199 mg/dL), or glycated haemoglobin (HbA1c) 39–46 mmol/mol (5.7%–6.4%) [16]. For obesity, the threshold was a BMI ≥30 kg/m^2^ [13]. For hypertension, the cutoff point was a systolic blood pressure ≥140 mmHg and diastolic blood pressure ≥90 mmHg [17].

For type 2 diabetes, individuals were considered to be newly diagnosed if they had a fasting capillary glucose level ≥6.99 mmol/L (≥126 mg/dL) or non-fasting capillary glucose level ≥11.1 mmol/L (≥200 mg/dL) [18]. At screening, controlled type 2 diabetes was defined as having a fasting capillary glucose level <7.21 mmol/L (<130 mg/dL) or non-fasting capillary glucose level <7.77 mmol/L (<140 mg/dL), and HbA1c <53 mmol/mol (<7%) [19, 20]. Uncontrolled obesity was defined as having a BMI >40 kg/m^2^. Uncontrolled hypertension was defined as having a systolic blood pressure >160 mmHg and/or diastolic blood pressure >100 mmHg. Individuals were considered to have uncontrolled type 2 diabetes if they had a fasting capillary glucose level >10.16 mmol/L (>183 mg/dL) or non-fasting capillary glucose level >11.1 mmol/L (>200 mg/dL).

A diagnosis gap in the continuum of care was defined as the proportion of individuals living with an NCD (obesity, hypertension, or type 2 diabetes) who did not have a prior diagnosis and were detected as having an NCD by MIDO COVID screening.

### Statistical analysis

A multivariable logistic regression model was used to analyse the relationship between the probability of infection and NCDs, adjusted by sex, age, and degree of marginalisation (a summary measure used in Mexico to describe populations according to factors such as housing, income, education, and population distribution) [21, 22]. Odds ratios (ORs) with 95% confidence intervals (CIs) were calculated. We tested the following variables for interaction using an α of 0.05: age, sex, and degree of marginalisation. We then estimated the probability of SARS-CoV-2 infection according to presence of NCDs and sociodemographic factors of age, sex, and degree of marginalisation. The probability of contagion was defined as IgG-positive results on serological testing (COVID-19 IgG-IgM Cassette, Hangzhou Biotest Biotech Co. Ltd., Zhejiang, China; or COVID-19 IgM/IgG Duo, SD Biosensor Inc., Suwon, Republic of Korea). Statistical analyses were performed using STATA 15 15.0 (StataCorp, College Station, Texas, USA).

## Results

**Fig 1:**
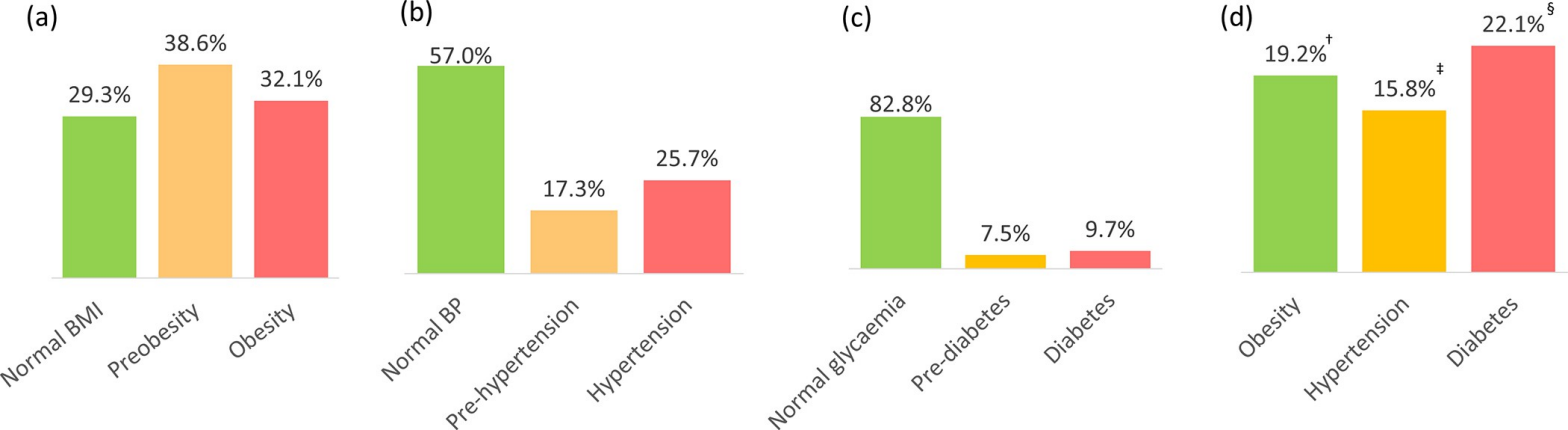
Prevalence of non-communicable diseases and pre-disease stages detected by MIDO COVID (N = 58,277): (a) obesity, (b) hypertension, and (c) diabetes. Percentages of patients previously aware of their condition are shown in (d). ^†^N = 18,718. ^‡^N = 14,950. ^§^N = 5,659. Abbreviations: BMI, body mass index; BP, blood pressure; MIDO, Integrated Measurement for Early Detection.

Of the 58,277 individuals screened, 60.5% were men and the majority (82.6%) were between the ages of 20 and 49 years (**Table 1**). The prevalence of obesity, hypertension, and type 2 diabetes detected by MIDO COVID was 32.1%, 25.7%, and 9.7%, respectively. In comparison, the percentages of individuals who self-reported these conditions (i.e., were previously aware they had the diagnosis) were 2.2%, 8.8%, and 4.5%, respectively. The prevalence of NCDs and pre-disease stages detected by MIDO COVID is shown in **Fig 1**. The prevalence of pre-obesity was 38.6%; pre-hypertension, 17.3%; and prediabetes, 7.5%. Of the 18,718, 14,950, and 5,659 individuals with obesity, hypertension, and type 2 diabetes, respectively, 19.2%, 15.3%, and 22.1% were considered as having an uncontrolled condition.

**Table 1:** Characteristics of screened population using MIDO COVID (June 26, 2020–December 31, 2020), by sex.

	Men	Women	Total
	N = 35,255	N = 23,022	N = 58,277
	(60.5%)	(39.5%)	(100.0%)
**Demographics**			
Age, years			
<20	1,372 (3.9)	1,187 (5.2)	2,559 (4.4)
20–29	8,757 (24.8)	6,126 (26.6)	14,883 (25.5)
30–39	11,498 (32.6)	8,055 (35.0)	19,553 (33.6)
40–49	8,572 (24.3)	5,112 (22.2)	13,684 (23.5)
50–59	4,652 (13.2)	2,402 (10.4)	7,054 (12.1)
≥60	404 (1.2)	140 (0.6)	544 (0.9)
**Lifestyle and risk factors**			
Smoking (yes, within past 12 months)	11,524 (32.7)	4,468 (19.4)	15,992 (27.4)
Lack of physical activity^a^	21,893 (62.1)	12,087 (52.5)	33,980 (58.3)
<6 hours of sleep per night	28,941 (82.1)	17,197 (74.7)	46,138 (79.2)
Parental history of type 2 diabetes	12,241 (34.7)	8,249 (35.8)	20,490 (35.2)
**Anthropometrics**			
Height, m (mean ± SD)	1.7 ± 0.1	1.6 ± 0.1	1.7 ± 0.1
Weight, kg (mean ± SD)	82.2 ± 16.4	68.1 ± 15.1	76.8 ± 17.4
Body mass index, kg/m^2^ (mean ± SD)	28.0 ± 5.2	27.2 ± 5.9	27.7 ± 5.5
Waist circumference, cm (mean ± SD)	97.7 ± 12.4	89.3 ± 12.7	94.4 ± 13.2
**Biomarkers**			
Systolic blood pressure, mmHg (mean ± SD)	129.8 ± 15.5	120.6 ± 14.9	126.2 ± 15.9
Diastolic blood pressure, mmHg (mean ± SD)	79.6 ± 10.9	74.3 ± 10.7	77.5 ± 11.1
Fasting capillary glucose, mg/dL (mean ± SD)	105.5 ± 28.8	102.0 ± 27.8	104.1 ± 28.5
Fasting capillary glucose, mmol/L (mean ± SD)	5.9 ± 1.6	5.7 ± 1.5	5.8 ± 1.6
Non-fasting capillary glucose, mg/dL (mean ± SD)	116.1 (39.0)	109.6 ± 30.9	113.5 ± 36.1
Non-fasting capillary glucose, mmol/L (mean ± SD)	6.4 ± 2.2	6.1 ± 1.7	6.3 ± 2.0

^a^Defined as less than 30 minutes of exercise/day, by self-report.

Data are shown as n (%) unless otherwise stated.

Abbreviations: MIDO, Integrated Measurement for Early Detection; SD, standard deviation.

The distribution of co-morbidities in individuals with NCDs is shown in **Table 2**. A total of 29,405 (50.5%) individuals did not have an NCD. Of the total population screened, 19,462 individuals (33.4%) had one chronic condition, and 9,410 (16.1%) had two or more chronic conditions. The proportion of individuals with only one NCD was the highest for obesity (18.6%) compared with hypertension (10.5%) and type 2 diabetes (4.4%), and the proportion of individuals with a combination of co-morbidities was the highest for obesity and hypertension (10.8%) compared with other co-morbidity combinations.

**Table 2:** Distribution of co-morbidities in screened subjects.

NCD	Men	Women	Total
	N = 35,255	N = 23,022	N = 58,277
	(60.5%)	(39.5%)	(100.0%)
Without NCD	16,367 (46.4)	13,038 (56.6)	29,405 (50.5)
Only obesity	5,636 (16.0)	5,184 (22.5)	10,820 (18.6)
Only hypertension	4,746 (13.5)	1,344 (5.8)	6,090 (10.5)
Only type 2 diabetes	1,723 (4.9)	829 (3.6)	2,552 (4.4)
Obesity and type 2 diabetes	292 (0.8)	258 (1.1)	550 (0.9)
Obesity and hypertension	4,588 (13.0)	1,715 (7.5)	6,303 (10.8)
Type 2 diabetes and hypertension	1,193 (3.4)	319 (1.4)	1,512 (2.6)
Obesity, type 2 diabetes, and hypertension	710 (2.0)	335 (1.5)	1,045 (1.8)

Abbreviation: NCD, non-communicable disease.

**Fig 2:**
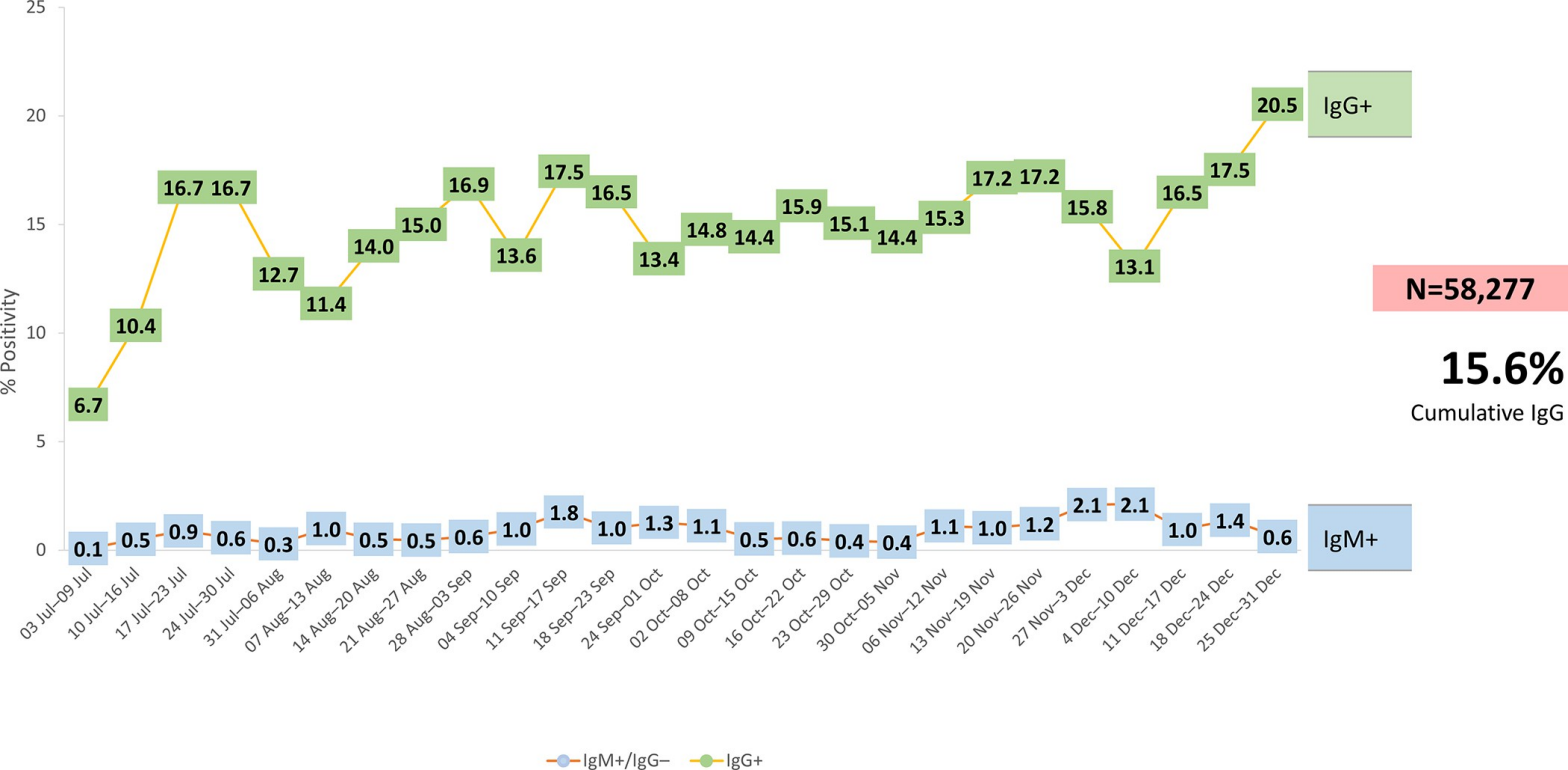
Weekly serological test positivity for SARS-CoV-2 by workplace testing (MIDO COVID; N = 58,277). Abbreviations: Ig, immunoglobulin; MIDO, Integrated Measurement for Early Detection.

**Fig 3:**
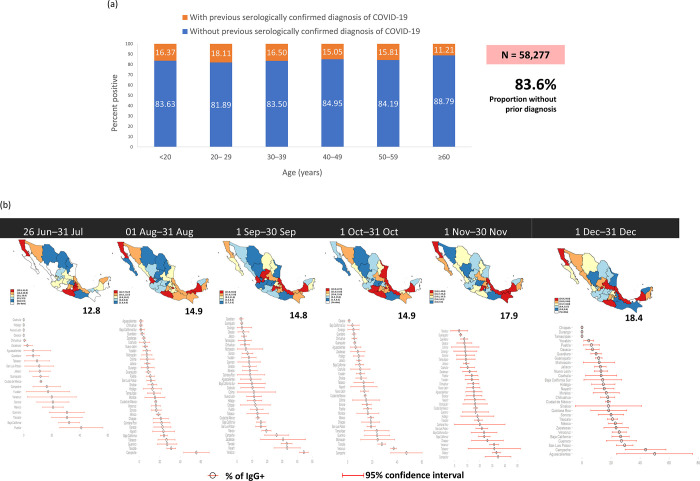
Proportion of IgG-positive tests according to previous diagnosis of COVID-19, stratified by age group (a), and monthly rates of IgG-positive tests according to Mexican state from June 26 to December 31, 2020 (b). States are colour-coded by quintile and data are the proportion of positive IgG test results with 95% confidence intervals.

The results of SARS-CoV-2 serological testing for IgG and IgM antibodies are shown in **Table 3**. Most individuals (83.4%) tested negative, 15.6% were either at the final phase of the acute stage of the disease or had a past infection, and 0.8% were in the early stage of the disease. **Fig 2** shows the weekly IgG and IgM positivity rates from July 3 until December 31. IgG positivity fluctuated from 6.7% to 20.5%. IgG positivity rates by prior diagnosis of COVID-19 in each age group are shown in **Fig 3A**. The rates of IgG-positive tests in those with a previous serologically confirmed diagnosis of COVID-19, subdivided by age categories, ranged from 11.21% in those aged ≥60 years to 18.11% in those aged 20–29 years. Age was not associated with IgG positivity according to previous diagnosis. **Fig 3B** illustrates monthly rates of IgG-positive tests at the Mexican state level. Although there was little change in the overall rate of positivity (i.e., at the national level), considerable changes were observed over time in individual states.

**Table 3:** Results of SARS-CoV-2 serological testing for IgG and IgM antibodies in asymptomatic subjects (N = 58,277).

Result	Interpretation	n (%)
IgM− / IgG−	Negative: has not been exposed to the virus	48,619 (83.4)
IgM+ / IgG−	Infection in early stages	487 (0.8)
IgM+ / IgG+	Final phase of the acute stage	2,375 (4.1)
IgM− / IgG+	Past infection	6,714 (11.5)
	Invalid test	82 (0.1)

Abbreviation: Ig, immunoglobulin.

The ORs for SARS-CoV-2 infection according to the presence of NCDs are shown in **Table 4**. The risk of infection was highest for those with combined obesity and type 2 diabetes (OR, 1.62; 95% CI, 1.30–2.01) and combined obesity, type 2 diabetes, and hypertension (OR, 1.58; 95% CI, 1.34–1.86), vs without an NCD (p<0.001 for both). The estimated probabilities of SARS-CoV-2 infection according to presence of NCDs and sociodemographic factors are presented in **Table 5**. The probabilities of infection were higher for all types and combinations of NCDs, across all sociodemographic categories, with the exception of the highly marginalised category, in which the lowest probability was estimated in the hypertension group. Obesity in particular contributed to a higher estimated probability of infection. Interestingly, a medium degree of marginalisation conferred the lowest probability of infection compared with both high and low marginalisation, among most combinations of NCDs.

**Table 4:** Odds ratios for SARS-CoV-2 infection according to presence of non-communicable diseases, sex, age, and marginalisation (N = 58,277).

	Odds ratio (95% confidence interval)	p-value
Without chronic condition	Reference	
Obesity	1.39 (1.31–1.47)	<0.001
Hypertension	1.33 (1.23–1.43)	<0.001
Diabetes	1.21 (1.08–1.35)	0.010
Obesity and type 2 diabetes	1.62 (1.30–2.01)	<0.001
Obesity and hypertension	1.42 (1.32–1.53)	<0.001
Type 2 diabetes and hypertension	1.46 (1.27–1.68)	<0.001
Obesity, type 2 diabetes, and hypertension	1.58 (1.34–1.86)	<0.001
Sex		
Men	Reference	
Women	0.97 (0.92–1.02)	0.201
Age		
≤20	Reference	
20–29	1.07 (0.94–1.21)	0.315
30–29	0.85 (0.75–0.96)	0.01
40–49	0.71 (0.63–0.81)	<0.001
50–59	0.71 (0.62–0.81)	<0.001
≥60	0.47 (0.34–0.63)	<0.001
Marginalisation		
Low	Reference	
Medium	0.37 (0.33–0.41)	<0.001
High	0.67 (0.61–0.74)	<0.001

**Table 5:** Estimated probability of SARS-CoV-2 infection according to presence of NCDs and by sociodemographic factors.

	Without NCD	Obesity	Hypertension	Type 2 Diabetes	Obesity and type 2 diabetes	Obesity and hypertension	Type 2 diabetes and hypertension	Obesity, type 2 diabetes, and hypertension
**Sex**								
Women	14.8 (14.1–15.6)	23.4 (21.7–25.0)	18.5 (15.8–21.3)	18.7 (15.1–22.2)	21.6 (14.6–28.5)	23.0 (20.1–25.8)	15.5 (10.4–20.6)	20.4 (14.5–26.3)
Men	16.9 (16.2–17.7)	20.6 (19.2–22.1)	22.4 (20.7–24.0)	17.7 (15.3–20.0)	26.2 (18.7–33.7)	22.4 (20.7–24.1)	22.9 (19.6–26.3)	24.1 (19.6–28.6)
**Age (years)**								
<30	17.6 (16.6–18.5)	24.7 (22.4–27.0)	29.8 (26.1–33.5)	16.3 (12.2–20.4)	25.4 (7.7–43.1)	31.3 (26.9–35.7)	25.5 (15.5–35.4)	25.3 (7.7–42.9)
30–49	14.9 (14.2–15.6)	21.6 (20.3–23.0)	19.6 (17.9–21.4)	19.0 (16.3–21.6)	26.8 (19.7–33.8)	21.8 (20.0–23.5)	21.3 (17.2–25.4)	26.2 (20.8–31.7)
50+	15.9 (14.0–17.9)	16.6 (13.8–19.3)	17.6 (14.7–20.4)	16.7 (12.7–20.7)	17.9 (10.1–25.6)	17.8 (15.1–20.5)	20.0 (15.8–24.3)	18.7 (13.9–23.4)
**Marginalisation**								
Low	18.2 (17.6–18.9)	23.5 (22.3–24.8)	22.8 (21.3–24.4)	19.9 (17.5–22.3)	25.2 (19.5–30.9)	23.5 (22.0–25.1)	22.3 (19.1–25.5)	24.1 (20.1–28.0)
Medium	6.4 (5.6–7.2)	11.1 (9.0–13.1)	11.3 (7.1–15.5)	7.1 (3.9–10.3)	9.5 (0.3–19.3)	11.3 (7.2–15.4)	17.4 (7.5–27.2)	11.9 (2.6–21.2)
High	11.8 (10.2–13.3)	19.7 (15.8–23.5)	10.8 (7.1–14.6)	16.5 (10.9–22.0)	27.0 (4.4–49.6)	16.6 (11.7–21.5)	14.4 (7.5–21.4)	15.2 (3.1–27.4)
**Overall**	16.0 (15.5–16.5)	21.9 (20.8–23.0)	21.5 (20.1–22.9)	18.0 (16.0–20.0)	24.0 (18.9–29.1)	22.5 (21.1–24.0)	21.3 (18.5–24.2)	22.9 (19.3–26.5)

Data are percentages with 95% confidence intervals.

Probabilities estimated after interaction logistic regression model.

Abbreviation: NCD, non-communicable disease.

## Discussion

The primary objective of our study was to characterise the burden of chronic diseases and COVID-19 in asymptomatic people in the workplace setting in Mexico. NCDs are known to have a strong influence on the severity and mortality of COVID-19 [3, 4, 23]. Therefore, the prevalence of chronic diseases in Mexico makes this population particularly vulnerable with respect to co-occurrence of NCDs and COVID-19. Unfortunately, the fragmented nature of health care systems in Mexico has negative effects on continuity of care, leading to unfavourable outcomes for patients.

The original MIDO system (prior to development of the COVID component) is already in use nationally in 1,169 primary care centres in Mexico and more than 2.4 million people have been assessed. This is an official system that reports the diagnoses to the National Chronic Disease Information System [12]. MIDO COVID is a comprehensive, person-centred strategy that responds to the need for an integrated plan in primary health care during the COVID-19 pandemic by facilitating the planning, surveillance, testing, and clinical management of COVID-19 and the chronic conditions related to the severity and mortality of the disease. By detecting pre-disease states, MIDO COVID allows for strategies to be implemented in a prophylactic manner, such that patients can improve their condition and do not subsequently progress from a pre-disease state to a diagnosis such as diabetes or hypertension. This has direct financial implications for health care systems, as achieving a lower incidence of NCDs leads to less expenditure on their treatment. Furthermore, by detecting pre-disease states, practitioners may be able to provide personalised follow-up. Metabolic control in particular is a cost-effective measure to avoid a wide variety of complications (e.g., vascular and neurologic). Furthermore, by identifying asymptomatic individuals who are IgM+ for SARS-CoV-2, we can provide immediate attention and efforts to isolate individuals to slow disease progression and avoid outbreaks, which also reduces financial burden on the health care system. Although COVID-19 vaccination was not available in Mexico at the time the present study was conducted, the ongoing MIDO COVID program will collect data on vaccination status as immunization programs are implemented.

MIDO COVID detected a critical gap between the self-reported health status of the individuals evaluated and the screening results. The prevalence of obesity, as detected by MIDO COVID, was approximately six times higher than that of self-reported obesity. In the UK, an increasing trend was found in the underassessment of overweight and obesity [24]. It was speculated that the social normalisation of overweight and obesity might have contributed to the misperception of weight status in the UK. This is also most likely the case in Mexico. Although the gaps between self-reported hypertension/diabetes and prevalence detected by MIDO COVID were smaller than the gap for obesity, these gaps remain a cause for concern. The early detection of NCDs and pre-disease states would facilitate the initiation of prevention and control strategies, including educational and awareness components for these conditions.

MIDO COVID identified that 10.8% of the population had both obesity and hypertension, a figure similar to the 9.9% prevalence reported nationwide [25]. It is normalised to be overweight or obese in Mexico; as such, it is not common for someone to perceive this as a disease state or something for which they should seek medical care. Underdiagnosed hypertension in Mexico is recognised as a public health problem nationwide; up to 40% of the population with hypertension do not know they have the disease [25]. Patients with hypertension usually do not present symptoms until an advanced stage, and in the absence of screening, the diagnosis of this disease is limited. MIDO provides a timely screening strategy that is expected to increase the early diagnosis of this disease.

MIDO COVID showed that among the 58,277 screened individuals, the prevalence of obesity, hypertension, and diabetes was 32.1%, 25.7%, and 9.7%, respectively, and among individuals with these conditions, 19.2%, 15.8%, and 22.1% had uncontrolled disease, respectively. These data were broadly comparable with data obtained from the original MIDO screening program [12], which included information gathered from 743,000 individuals in primary health care settings throughout Mexico, in which the prevalence of obesity, hypertension, and diabetes was 29.4%, 13.5%, and 9.9%, respectively. Although the prevalence of hypertension identified by MIDO screening was considerably lower than that of MIDO COVID (13.5% vs 25.7%), there was a high prevalence of pre-hypertension (19.2%) identified by MIDO. Interestingly, the sociodemographic characteristics of these study populations differed in several key ways: the MIDO COVID population was 60.5% male vs 30.5% in MIDO, and the age distribution was considerably younger in MIDO COVID, reflecting the fact that the screening was conducted in the workplace setting. Although mean BMI was not different between populations, history of smoking and parental history of diabetes were considerably higher in the present study.

Achieving proper control of NCDs, particularly obesity and cardiometabolic disease, might make patients less vulnerable to COVID-19 complications [5]. As MIDO COVID is performed on asymptomatic subjects, it is not considered an acute diagnostic strategy for COVID-19. However, because it has been estimated that at least 50% of SARS-CoV-2 transmission occurs via asymptomatic individuals [26], detection of asymptomatic infections as well as early identification of comorbidities related to COVID-19 severity is critically important. It should be noted that at the time of writing, a direct causal relationship between NCDs and risk of SARS-CoV-2 infection had not been established, although we found that the risk of infection was highest for those with combined obesity and type 2 diabetes and combined obesity, type 2 diabetes, and hypertension, compared with individuals without NCDs. Regarding the high rates of comorbidities among both high- and low-marginalized groups, we speculate that the rural vs urban lifestyle may contribute to the development of NCDs in each group in different ways. For example, highly marginalized groups are typically associated with rural areas in which there are barriers to accessing timely, high-quality health care. Such individuals with a lack of access are less likely to seek interventions or preventative care, potentially leading to a higher rate of NCDs. In the case of low marginalization, the dynamics of urban areas (easy access to nutrient-poor fast food, fewer opportunities to maintain a physically active lifestyle, crowding and other high-stress situations) may be the key factors.

The results of SARS-CoV-2 serological testing for IgG and IgM antibodies showed that most of the screened population (83.4%) had not been exposed to the virus. Importantly, the population undergoing screening is asymptomatic; thus, MIDO COVID can help put measures in place to prevent infection as well as control NCDs in those affected. Furthermore, MIDO COVID allows for early isolation of individuals in the active phase of COVID-19 (early or final phase of the disease) and prioritises metabolic control for those with NCDs; it may also identify the population that has been in contact with the virus.

Our geographic analysis indicated that although the national rate of IgG positivity did not vary greatly over the study period, considerable differences were observed in individual states. This cannot be interpreted as a reflection of the true prevalence, because the largest number of tests are carried out in locations with outbreaks. Nevertheless, these data allow us to verify whether health care services are adequate at a given time and location.

The present study has some limitations, including the possibility of selection bias. The results of MIDO COVID are not representative of the general Mexican population. Fasting plasma glucose and oral glucose tolerance tests, the gold standard tests for diagnosing type 2 diabetes [18], were not used.

In conclusion, the detection of various co-morbidities is essential to identify individuals who are especially vulnerable to COVID-19, particularly because patients with NCDs are at elevated risk for complications. In Mexico, obesity is normalised at the social level, and a critical gap was identified between self-reported health status and the presence of obesity, hypertension, and type 2 diabetes. A considerable proportion of the screened population was unaware of their health status; MIDO COVID may help educate and sensitise such people about NCDs and their association with the severity and mortality of COVID-19. MIDO COVID is based on the MIDO strategy, which currently operates in health centres throughout Mexico. Our research suggests that MIDO COVID could be applied in other workplace settings, schools, or large social gatherings; as such, this strategy can promote equity in access to health care resources and relevant services for as many individuals as possible during this pandemic, which may in turn lead to improvements in prevention and control of outbreaks [27]. Finally, at the time of this study, there was no access in Mexico to effective vaccines against SARS-CoV-2. Thus, the data obtained through MIDO COVID may serve to aid decisionmakers in prioritising the most vulnerable populations for immunisation programmes, once they become available.

## Supporting information

S1 File MIDO COVID screening protocol. (PPTX)

S2 File MIDO COVID questionnaire (translated from Spanish). (DOCX)

S3 File De-identified dataset. (XLSX)
