# Supplementary material for: MIDO COVID: A digital public health strategy designed to tackle chronic disease and the COVID-19 pandemic in Mexico
Source: PLoS One. 2022 Nov 17;17(11):e0277014. doi: 10.1371/journal.pone.0277014 (PMC9671410; doi:10.1371/journal.pone.0277014)
Supplement: S1 File — (PPTX) [file pone.0277014.s001.pptx]

## Slide 1
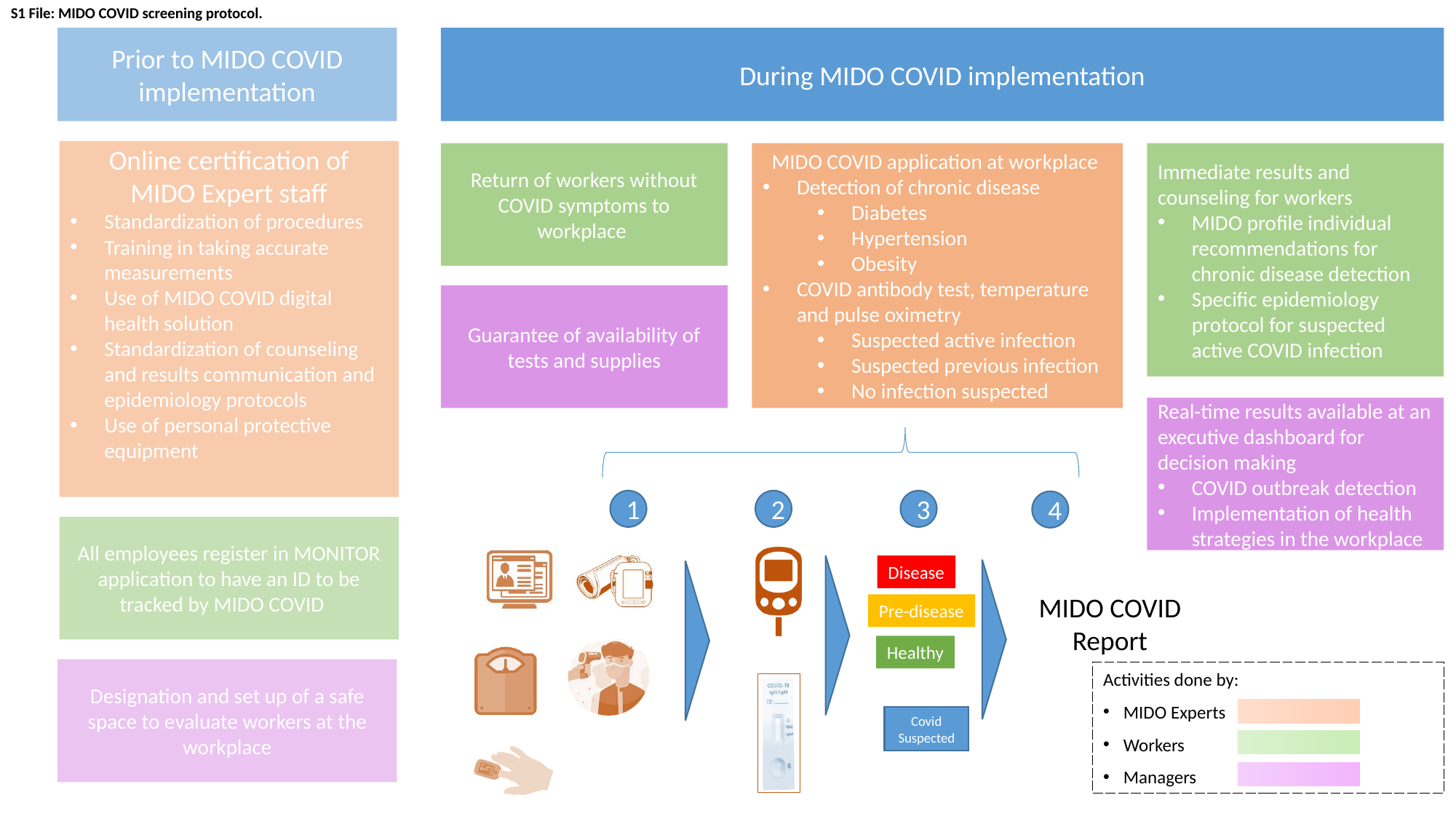

S1 File: MIDO COVID screening protocol.
Prior to MIDO COVID implementation
During MIDO COVID implementation
Online certification of
MIDO Expert staff
Standardization of procedures
Training in taking accurate measurements
Use of MIDO COVID digital health solution
Standardization of counseling and results communication and epidemiology protocols
Use of personal protective equipment
Return of workers without COVID symptoms to workplace
MIDO COVID application at workplace
Detection of chronic disease
Diabetes
Hypertension
Obesity
COVID antibody test, temperature and pulse oximetry
Suspected active infection
Suspected previous infection
No infection suspected
Immediate results and counseling for workers
MIDO profile individual recommendations for chronic disease detection
Specific epidemiology protocol for suspected active COVID infection
Guarantee of availability of tests and supplies
Real-time results available at an executive dashboard for decision making
COVID outbreak detection
Implementation of health strategies in the workplace
1
2
3
4
All employees register in MONITOR application to have an ID to be tracked by MIDO COVID
Disease
MIDO COVID Report
Pre-disease
Healthy
Designation and set up of a safe space to evaluate workers at the workplace
Activities done by:
MIDO Experts
Workers
Managers
Covid Suspected
