# Supplementary material for: MIDO COVID: A digital public health strategy designed to tackle chronic disease and the COVID-19 pandemic in Mexico
Source: PLoS One. 2022 Nov 17;17(11):e0277014. doi: 10.1371/journal.pone.0277014 (PMC9671410; doi:10.1371/journal.pone.0277014)
Supplement: S2 File — (DOCX) [file pone.0277014.s002.docx]

# **Supporting information**

## **S2 File. MIDO COVID Questionnaire (translated from Spanish)**

**1. Personal information:**

| MONITOR ID: | | | |
| --- | --- | --- | --- |
| Full name: | | | |
| Date of birth: | Place of birth (state) | Sex:  M F | Pregnant:  Yes No |
| Residence (state): |  | Mobile telephone: | |
| Email address: | | | |

**2. Risk factor questionnaire:**

| # | Answer the following questions: | | |
| --- | --- | --- | --- |
| 1 | Has your doctor told you that you have diabetes? | Yes | No |
| 2 | Has your doctor told you that you have hypertension? | Yes | No |
| 3 | Have you had elevated glucose levels recently? | Yes | No |
| 4 | Have you had a heart attack or a stroke? | Yes | No |
| 5 | Do/did either of your parents have diabetes mellitus? | Yes | No |
| 6 | Do/did any of your siblings have diabetes mellitus? | Yes | No |
| 7 | Do you smoke or have you smoked in the past 12 months? | Yes | No |
| 8 | Do you get little or no exercise? | Yes | No |
| 9 | Do you regularly sleep for at least 6 continuous hours? | Yes | No |

**3. Basic measurements:**

| Weight: ________________ Height: ________________ Waist: ________________  **Blood pressure measurement:**  1^st^ Measurement: ________________ 2^nd^ Measurement: ________________  **Capillary glucose measurement:**  Fasting: ⃝ Casual ⃝ Result:_____________ |
| --- |

**4. COVID-19 questionnaire:**

| **Have you been diagnosed with COVID-19?** Yes: ⃝ No: ⃝  Temperature: __________________ Oxygen saturation: __________________  Was a serological test performed? Yes: ⃝ No: ⃝  Type of test: ___________________ Date of test: ________________________  Test result: ⃝ Invalid/inconclusive ⃝ IgM+/IgG− ⃝ IgM−/IgG+  ⃝ IgM−/IgG− ⃝ IgM+/IgG+ |
| --- |
